# Phage Biosensor for the Classification of Metastatic Urological Cancers from Urine

**DOI:** 10.3390/life14050600

**Published:** 2024-05-08

**Authors:** Vilhelmiina Juusti, Antti Rannikko, Anu Laurila, Maria Sundvall, Pekka Hänninen, Janne Kulpakko

**Affiliations:** 1 Laboratory of Biophysics and Medicity Research Laboratories, Institute of Biomedicine, Faculty of Medicine, University of Turku, Tykistökatu 6A, 20520 Turku, Finland; 2 Aqsens Health Ltd., Itäinen Pitkäkatu 4B, 20520 Turku, Finland; 3 Department of Urology, Helsinki University, Helsinki University Hospital, 00280 Helsinki, Finland; 4 Department of Oncology, Turku University Hospital, PL52, 20521 Turku, Finland; 5 Cancer Research Unit, Institute of Biomedicine, FICAN West Cancer Center Laboratory, University of Turku, Turku University Hospital, Kiinamyllynkatu 10, 20520 Turku, Finland

**Keywords:** phage, biosensor, phage biosensor, chemical sensor, metastatic cancer, urological cancer, liquid biopsy, urine, liquid crystal

## Abstract

Most of the annual 10 million cancer-related deaths are caused by metastatic disease. Survival rates for cancer are strongly dependent on the type of cancer and its stage at detection. Early detection remains a challenge due to the lack of reliable biomarkers and cost-efficient screening methods. Phage biosensors can offer a solution for early detection using non-invasive liquid biopsies. Here, we report the first results of the bifunctional phage biosensor to detect metastatic urological cancers from urine. A dye-sensitized phage library was used to select metastasis-related phage binders. After selection rounds, the most promising phage candidate was used to classify metastatic cancer from controls. Additionally, we applied one chemical sensor (phenoxazine non-fluorescent dye) to classify cancer from urine. A statistical significance (*p* = 0.0002) was observed between metastatic and non-metastatic cancer, with sensitivity of 70% and specificity of 79%. Furthermore, the chemical sensor demonstrated significance in detecting cancer (*p* < 0.0001) with a sensitivity of 71% and a specificity of 75%. Our data suggest a new promising field for urine biomarker research, and further evaluation with prospectively collected samples is ongoing. In conclusion, we report, for the first time, the potential of a chemical- and phage-based biosensor method to detect metastatic cancer using urine.

## 1. Introduction

Cancer is a notable health challenge of the 21st century. Despite vast efforts in research, detection, and treatment, it was still among the leading causes of death in 2022 [1]. Most cancer-related morbidity and mortality is caused by metastases [2,3]. The formation of metastases is a process in which a malignant cell invades and migrates from the primary origin to surrounding tissue or distant secondary organs and establishes a macroscopic secondary focus, commonly known as metastasis. Metastasis formation is regulated by a complex interplay of genetic and epigenetic alterations in the cell, shaping cell morphology and phenotype [4,5]. Cells can metastasize even before the primary tumors are detectable with conventional detection methods [3], causing late detection and poor prognosis.

Moreover, there is a lack of clinically relevant biomarkers that could be used for early detection of metastatic cancer via liquid biopsies. Only a handful of metastasis-related serum biomarkers have demonstrated clinically relevant associations with advanced disease and metastases, such as CA-125 for ovarian cancer and CA 15-3 and CA 27.29 for breast cancer. Many investigational biomarkers, such as vascular endothelial growth factor (VEGF), chemokines, cytokines, heparanase, matrix metalloproteinases, and miRNAs have been linked to early metastatic processes [6,7,8]. In addition, increased levels of circulating tumor DNA (ctDNA) indicate metastatic disease and decreased survival [9,10].

Multi-cancer early detection (MCED) tests have gained attention as promising screening tools, even for metastatic cancer. They combine the molecular analysis of tumor-related markers using liquid biopsies, like urine, and tissues with artificial intelligence. Nevertheless, urine as a liquid biopsy has been studied limitedly for the detection and classification of various cancers [11]. It can offer a new source to find relevant, yet unknown, biomarkers related to metastases. Evidently, the same biomarkers present in serum can be found in urine with different levels [12,13,14]. Serum is known for Its outstanding buffering properties, which can hinder the detection of biomarkers in low quantities. Hence, urine can contain biomarkers from urological malignancies that are excreted only in it, or these biomarker concentrations may be higher in urine than in serum. It can also facilitate easier sampling [15] and expand cancer screening availability to a broader population. However, few molecular tests are currently sensitive enough to detect relevant biomarkers in urine.

Cancer-related biomarkers are usually proteins, and therefore, immunoassays have been the method of choice for detecting them. In recent years, biosensors with various biorecognition elements have garnered attention [16,17,18]. When new biosensor technologies are developed, their performance must be compared to clinical diagnosis made with the current gold standards and clinical guidelines. Test sensitivity and specificity have to meet, and preferably exceed, those of the corresponding current methods. Additionally, modern cancer detection tests should be accurate, affordable, user-friendly, quick, and tolerant to environmental factors. Foremost, the clinical applicability of the test has to be good for integrating it into clinical practices.

From different analytical biosensors, phage biosensors have high precision and tunability toward different targets [19,20]. Furthermore, they are easily linked to different transducer options and have low production costs and a long shelf-life [21]. The structure and physicochemical properties of phages make them suitable for detection purposes, as they are tolerant to harsh conditions, such as different ranges of pHs, temperatures, and dissolvents. Therefore, they are one option to construct modern biosensors for biomarker detection. Another advantage is that phage biopanning is a well-known and straightforward process in which the best binder is selected for further tests. Essentially, a desired variant with the highest affinity with the analyte molecule can be chosen from a wide pool of phages via affinity selection [19].

We have demonstrated how phage biosensors can be designed to detect a single biomarker protein and, more importantly, to classify urine liquid biopsies, such as distinguishing lethal prostate cancer from non-lethal cancer [22]. Later, the biophysical properties of phage biosensors were further studied [23]. Our main finding was that the liquid crystalline (LC) behavior of phages produced the detection event. Biosensors based on this kind of molecular ordering have been reviewed recently [18]. However, biosensors enabling the LC behavior of bifunctional phages have not been reported before.

**Figure 1:**
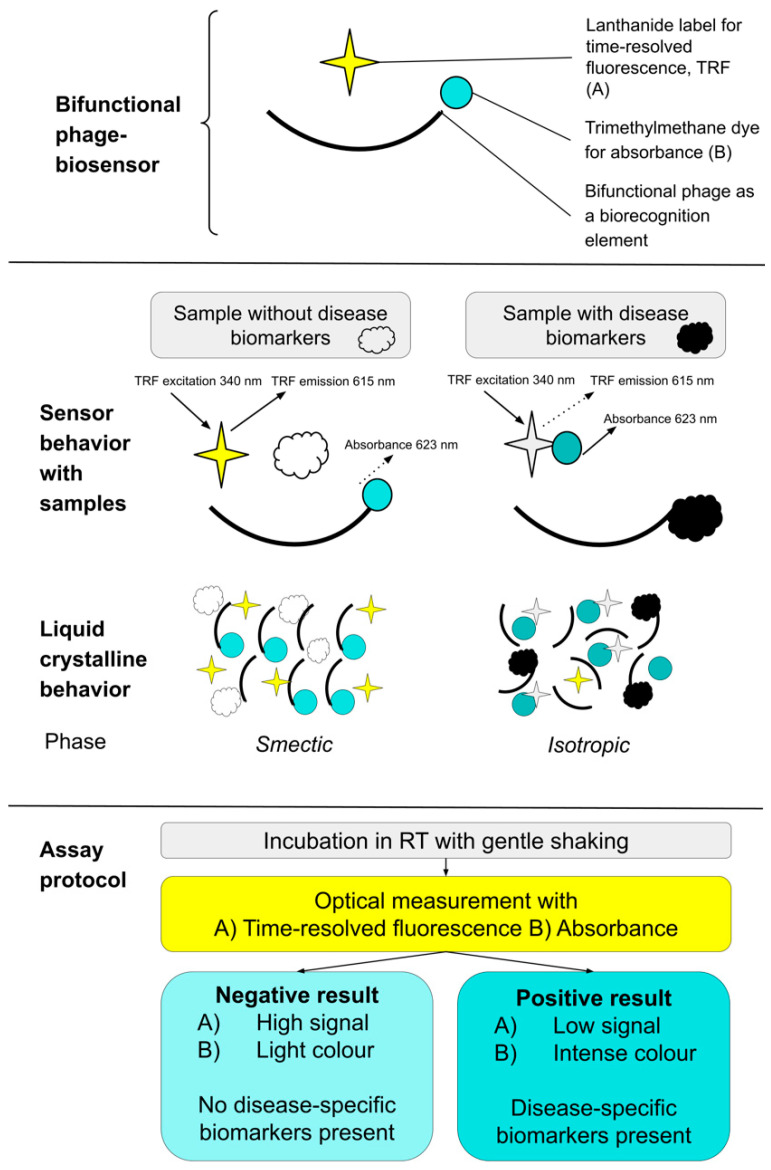
Schematic presentation of bifunctional phage biosensor method. The biosensor consists of three components, including the bifunctional phage (affinity with the dye and disease-specific biomarkers), lanthanide label, and trimethylmethane dye. The biosensor behaves differently with a negative sample not containing the disease-specific biomarkers and a positive sample containing them. The result is measured with TRF and/or absorbance. In more detail, the detection is based on the liquid crystalline (LC) behavior of the bifunctional phage. Depending on the sample, phages either order themselves with dye molecules (negative sample) or their organized structure is contravened (positive sample).

The detection system is based on the bifunctional phage interacting with the disease-related biomarkers, lanthanide label and trimethylmethane dye. The phage interacts with the dye molecule when the biomarkers are not present (i.e., negative sample). Therefore, the lanthanide label is not quenched and emits a time-resolved fluorescence (TRF) signal. Simultaneously, the color changes from light green because of the phage–dye interaction. When a sample contains the biomarkers (i.e., positive sample) the phage interacts with them, and the dye molecule quenches the lanthanide label. This causes a decrease in the measured TRF signal, and the color remains or darkens in the solution because the phage is not interacting with the dye. In more detail, the detection is based on the LC behavior of the phage. With a negative sample, phages order themselves with dye molecules into an organized smectic phase. With a positive sample, the smectic phase is contravened by specific biomarkers that phages have affinities with, and the LC phase changes to a disorganized isotropic phase. The components, function, interpretation of absorbance, and TRF results of this biosensor technology are presented in Figure 1. In summary, we demonstrate a more refined phage biosensor method for distinguishing metastatic urological cancers from non-metastatic ones. It provides a means to categorize malignancies without prior knowledge of specific biomarkers that can overcome the constraints of conventional techniques.

## 2. Materials and Methods

### 2.1. Clinical Samples

A cohort of 74 urine samples was used in this study (Table 1). Cancerous samples (n = 53) were collected from patients with prostate, kidney, and other cancers (pancreas, bladder, penis, testis, retroperitoneal). Control samples (n = 21) were collected from healthy volunteers without previous cancer diagnoses. Ethical approvals for the use of urine samples and clinicopathological data were obtained from the Institutional Ethics Committee of the Hospital District of Helsinki Uusimaa (HUS/850/2017 for the DEDUCER trial) and from the Ethical Committee of the Hospital District of Southwest Finland (ETMK 29/1801/2022 for the BIOURICA study). The DEDUCER trial is a prospective clinical trial in which participants are patients with urological malignancies in the HUS Helsinki University Hospital. PCa diagnostics was based on prostate MRI as a triage test, followed by targeted ± systematic prostate biopsies. BIOURICA is an ongoing prospective research study for detecting cancer biomarkers with phage biosensors in collaboration with TYKS Turku University Hospital and Aqsens Health Ltd. Cancer diagnostics and malignancy was confirmed with a biopsy and pathological analysis and, in metastatic cases, with imaging methodology (CT, MRI, or PET). The ethical standards of the Helsinki Declaration were followed, and written informed consent was obtained from all study participants. All relevant guidelines and regulations were followed while performing the experiments.

### 2.2. Sample Preparation

Original urine samples were centrifuged with a rotational speed of 8000 rpm for 10 min to separate the solid material from the liquid. Supernatants were diluted 1:10 (for biopanning) and 1:50 (for assays) with 0.9% saline solution. The pH of each original sample was measured using a SevenCompact^TM^ S220 pH meter (Mettler Toledo, Columbus, OH, USA) to exclude the effect of pH on classification with sensors.

### 2.3. Biopanning Bifunctional Phages

We suggest here an improvement to the second stage of the biopanning method based on our previously published protocol [22,23]. The first stage of biopanning was performed to produce a primed M13 phage library from a commercial combinatorial library containing random 12-mer peptides (~10^9^ unique sequences) fused to a minor coat protein (pIII) of the M13 phage. A trimethylmethane dye (brilliant green) was attached to lignin chips. Lignin was used because it binds the dye better than polystyrene. Phages from a commercial library were introduced to dye molecules, and those with affinities with the dye molecules were selected and amplified. Amplification was performed through infection with *E. coli* strain K12 ER2738, and amplified phages were collected using centrifugation with a rotational speed of 15,000 rpm for 10 min.

Then these amplified dye-sensitized phages were used as a primed library to select possible phage binders with affinities with metastasis biomarkers from clinical urine samples. The biopanning conditions, like absorbents and sample processing (dilutions, pools, fractionalizing), were modified to enhance the selection of the most specific binders able to distinguish between metastatic and non-metastatic samples. First, an aliquot of the primed phage library was biopanned directly against pools (n = 7) of urine dilutions (1:10), both metastatic and non-metastatic immobilized to lignin chips. Two cycles of this affinity selection were repeated with 8 washes with saline solution. Second, other aliquots of the primed library were biopanned against the size exclusion-fractionated (illustra^TM^ NAP-10 column with Sephadex^TM^ G-25 DNA grade, GE HealthCare, Chicago, IL, USA) metastatic pool (n = 7). Fractionating was performed by adding 1 mL of diluted pool to a column and washing it with 3 mL of saline solution. The fractions were collected in a volume of ~20 µL and immobilized to a polystyrene surface before biopanning stage 2. Then, two cycles of selection were repeated, with 6 washes with saline solution for size-fractionated pools. Each biopanning cycle yielded a biosensor candidate that could be used for another biopanning round.

**Figure 2:**
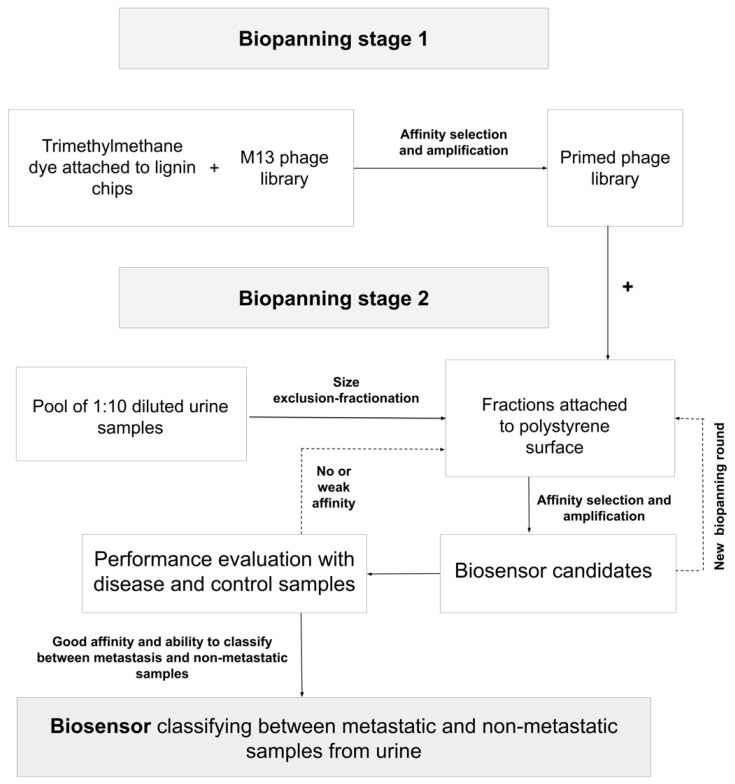
Schematic presentation of the biopanning strategy for phage biosensors detecting metastatic cancer from fractionated urine. In the first biopanning stage, the primed phage library is prepared with affinity selection toward trimethylmethane dye. This library is used in the second biopanning stage to perform affinity selection toward size exclusion-fractionated diluted urine samples. Biosensor candidates (phages after affinity selection) are amplified and tested with clinical disease and control samples. In instances in which there is no or weak affinity, the process of secondary affinity selection is iterated or repeated until a satisfactory classification is attained. Then, a biosensor is ready to be used in assays to distinguish between metastasis and clinical samples.

A total of 29 biosensor candidates was found after these two selection rounds. The number of selection rounds was chosen based on our experimental studies to reach satisfactory affinities with both the dye and target biomarkers with the selected phages to have optimal conditions for the LC behavior in the biosensor. The amounts of biosensor components (phage, dye, sample dilution) were optimized in the performance evaluation step. In particular, the phage amount affects the LC behavior of the biosensor [23]. The candidates were further tested according to their response to the disease (metastatic cancer) and control samples with the protocol described below (2.5 assays with the biosensor). From these candidates, the best one, having the ability to classify between metastatic and non-metastatic samples, was used as a bifunctional phage together with a control phage (primed library) in the biosensor assays. The chosen biosensor has the largest statistical significance in classification and difference from the results with the primed library. In case biosensor candidates would not have an affinity or have a weak affinity with target samples, candidates were biopanned again, targeting fractionated samples, and optimized and tested again. The main stages for the entire biopanning process with fractionated urine samples are presented in Figure 2.

### 2.4. Assays with the Chemical Sensor

A previously used [22] chemical, a phenoxazine non-fluorescent dye 7-hydroxy-3H-phenoxazin-3-one-10-oxide sodium salt (Sigma-Aldrich, St. Louis, MO, USA) related to the stage of prostate cancer, was tested with the sample cohort. The aim was to study whether the chemical is classifying lethal prostate cancer only or if the interaction is rather generic with cancerous or metastatic cancer samples. The assay was performed by adding the chemical (185 µM) to each microplate well (ThermoFisher, Waltham, MA, USA). Then, 100 µL of the processed urine sample was added to the wells in three replicates. Finally, a lanthanide label was added to each well (3.7 µM of EuCl_3_ · 6 H_2_O, 2.2 µM NSC 42,790 and 2.2 µM OP(C_8_H_17_)_3_, Sigma-Aldrich, St. Louis, MO, USA). An europium ion emits TRF when combined in the label solution with organic antenna molecules assisting in the energy transfer. The TRF (excitation 340 nm and emission 615 nm, with a 400 μs window after a 400 μs delay time) was measured after 15 min in the wells. In addition to TRF measurement, absorbance (λ = 610 nm) was measured after 5 min. A Spark multimode microplate reader (Tecan, Switzerland) was used in the measurements. Before and between the measurements, the plate was incubated in RT with shaking (450 rpm).

### 2.5. Assays with the Biosensor

Assays with a bifunctional phage and a control phage (primed library) were performed in a largely similar fashion with a chemical sensor. First phage solution (4.0 × 10^9^ pfu/mL) and trimethylmethane dye in brilliant green (1 mM) were added to each microplate well. Then, 100 μL of the processed urine sample was added to the wells in three replicates. Absorbance (λ = 623 nm) was measured every 5 min from 2 min to 180 min. Before and between measurements, the plate was incubated in RT with shaking. The most statistically significant measurement (*p* < 0.05) was chosen to be analyzed in more detail and presented in Section 3.

### 2.6. Data Analysis

To estimate the classification accuracy of the biosensor and chemical sensor assays toward cancer in general and metastatic cancer from non-metastatic cancer and controls, we compared our assay results to the clinical status of each study participant. The urine dilutions of each original sample were used in the assays in three replicates, which were individually measured, and a mathematical median of the three measurements was estimated and used in all analyses.

**Figure 3:**
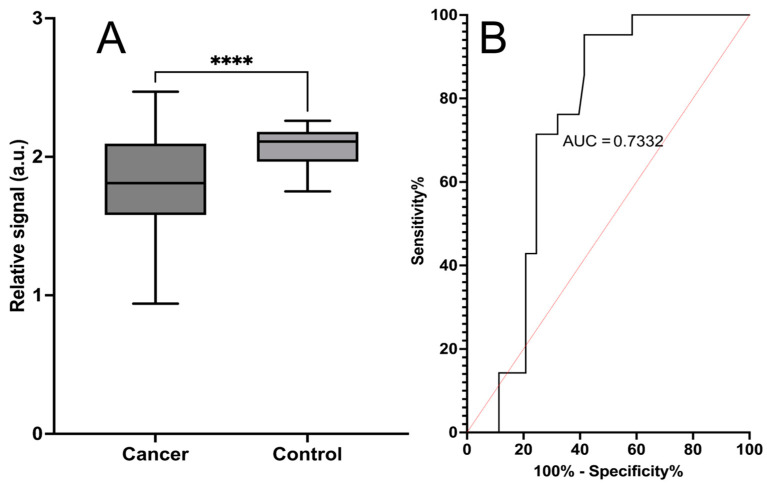
Cancer detection with a chemical sensor based on a phenoxazine non-fluorescent dye. Absorbance (λ = 610 nm) was measured after 5 min of incubation. Plot (**A**) displays the signal mean of three replicate measurements, with SEM as error bars between the cancer samples (n = 53) and non-cancerous control samples (n = 21). **** Statistical significance (*p* < 0.0001) between cancerous and non-cancerous samples was reached. Plot (**B**) presents the ROC curve, with an area under the curve of 0.73. The calculated sensitivity of the assay is 71%, and the specificity is 75%.

**Figure 4:**
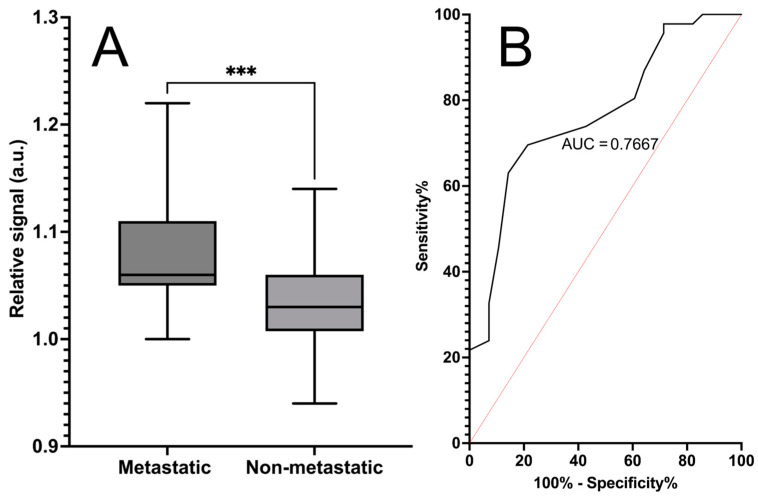
Detection of metastatic cancer using a phage biosensor based on bifunctional phages. Absorbance (λ = 623 nm) was measured with a developed metastasis-specific biosensor and control biosensor. Plot (**A**) displays the standardized signal mean of three replicate measurements, with SEM as error bars between the metastatic samples (n = 28) and non-cancerous control samples (n = 46). Signals for each sample were standardized by dividing the signals of the bifunctional biosensor by the signals of the control biosensor. *** Statistical significance (*p* = 0.0002) between metastatic and non-metastatic samples was reached. Plot (**B**) presents the ROC curve, with an area under the curve of 0.77. The calculated sensitivity of the assay is 70%, and the specificity is 79%.

With the biosensor, the median of each sample was divided by the median of the same sample measured with a control biosensor (primed library), and the ratio for each sample was used in the final analyses. All results were analyzed using Prism 9.4. To identify outliers, the method ROUT was used, with the parameter “Q” set to “definitive outliers” (0.1); no outliers were detected (Figure 2, Figure 3 and Figure 4).

## 3. Results

We measured a cohort of 74 urine samples, including metastatic cancer, non-metastatic cancer, and control samples, with one chemical sensor and biosensor method. In addition, we studied the effects of pH and dilution on the classification.

### 3.1. Detection of Cancer with the Chemical Sensor

The chemical sensor was selected based on screening of our cancer-associated chemical sensors (n = 4) that previously indicated selectivity between cancerous and control samples from urine. The chemical sensor used, phenoxazine non-fluorescent dye, has been used in our previous study related to lethal prostate cancer detection [22] with TRF. Herein, we report its usefulness in cancer detection with absorbance reading instead of TRF for the first time. Absorbance from the chemical sensor was measured and revealed significantly different values (*p* < 0.0001) between cancerous (n = 55) and non-cancerous control (n = 21) samples, with an area under the curve (AUC) of 0.73. Hereby, the calculated sensitivity for the sensor was 71% (95% Cl 50–86%), and the specificity was 75% (95% Cl 62–85%). These results are shown in Figure 3. TRF was measured after 15 min of incubation and revealed significantly different values (0.0066) between cancerous and non-cancerous control samples, with an area under the curve (AUC) of 0.70. The calculated sensitivity for the sensor was 62% (95% Cl 41–79%), and the specificity was 72% (95% Cl 58–82%).

### 3.2. Classification of Metastatic Cancer from All Non-Metastatic Samples

Our biosensor selection and analysis process has significantly improved after our last study [22]. In this study, biopanning was conducted following the size-exclusion fractionation of urine pools for two primary reasons (Figure 1 and Figure 2). Firstly, the size-exclusion gel purification process reduces the presence of potential biomarker-masking molecules within the sample. Secondly, the size-exclusion column aids in the separation of the sample into distinct molecular groups, facilitating the identification of potential biomarkers. Additionally, the biosensor candidates were tested at multiple time points during a 3 h period to reveal the optimal time point for classification. In the data analysis, we found that standardizing the measurement results with a developed biosensor to the results with a primed library increased the classification power. Earlier, we used diluted urine pools for biopanning, measured only a few time points to find the proper biosensor, and used non-standardized measurement results in the analysis. Altogether, 29 biosensor candidates were developed and tested during this study. Among these, the best one with the highest affinity with metastatic cancer from urine was chosen to assay with the whole sample cohort.

The chosen biosensor revealed significantly different absorbance values (*p* = 0.0002) between metastatic cancer and non-metastatic cancer samples (both non-metastatic cancer and control samples), with an AUC of 0.77. The calculated sensitivity for the sensor was 70% (95% Cl 55–81%), and the specificity was 79% (95% Cl of 60–90%) in metastatic cancer detection. These results are shown in Figure 4.

### 3.3. Classification of Metastatic Cancer and Non-Metastatic Cancer Samples

**Figure 5:**
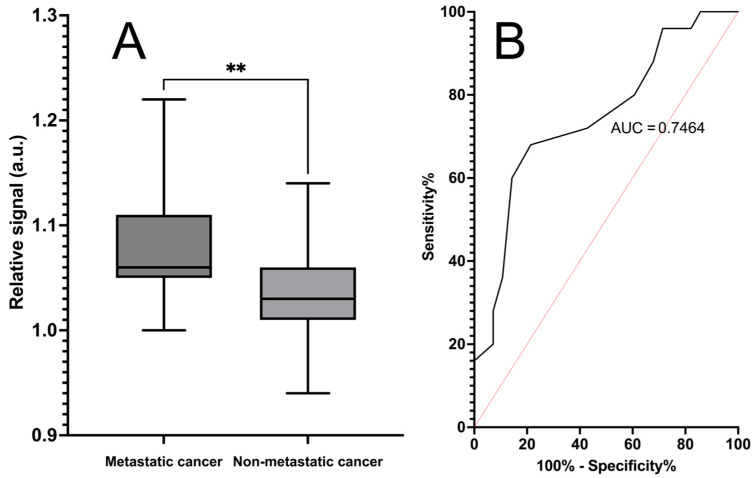
Classification of metastatic cancer and non-metastatic cancer samples with the phage biosensor based on bifunctional phages. Absorbance (λ = 623 nm) was measured after 180 min of incubation with the developed metastasis-specific biosensor and control biosensor. Plot (**A**) displays the standardized signal mean of three replicate measurements, with SEM as error bars between the metastatic samples (n = 28) and non-metastatic cancer samples (n = 25). Signals for each sample were standardized by dividing signals of the biosensor by the signals of control biosensor. ** Statistical significance (*p* = 0.0017) between metastatic and non-metastatic samples was reached. Plot (**B**) presents the ROC curve with an area under the curve of 0.75. The calculated sensitivity of the assay is 68%, and the specificity is 79%.

Additionally, we analyzed the classification power of the bifunctional biosensor together with the control biosensor between metastatic and non-metastatic samples when the non-cancerous control samples were excluded from the sample set. The biosensor was able to classify between these two groups and showed a statistically significant difference (*p* = 0.0017), although the area under the curve decreased from 0.77 to 0.75. Herein, the calculated sensitivity decreased to 68% (95% Cl 48–83%), but the specificity remained at 79% (95% Cl 60–90%). These results are shown in Figure 5.

### 3.4. Kinetic Color Change of Phage Biosensor in Detection of Metastasis

**Figure 6:**
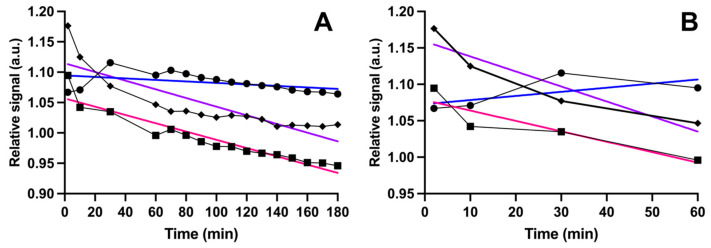
Kinetic behavior of biosensor with samples. The blue line shows the slope for relative color (absorbance) change as a function of time with a metastatic sample (dots), the purple line shows the slope for a non-metastatic (rhombus), and the pink line shows the slope for a control sample (square). Plot (**A**) shows that the slope of linear regression during 180 min was smaller for the metastatic sample (−0.0001235) than for the non-metastatic (−0.0007139) and non-cancerous control samples (−0.0006815) (**A**). Plot (**B**) shows that during the first 60 min of detection, the slope was positive for the metastatic sample (0.0005643) and negative for the non-metastatic (−0.002061) and control samples (−0.001428).

We found that biosensor kinetic behavior differs between metastatic and non-metastatic samples. The color formation (change in absorbance) as a function of time in detection was analyzed for a few individual samples. During the whole measurement (180 min), the slope of simple linear regression was −0.0001235 for the metastatic sample, −0.0007139 for the non-metastatic cancer sample, and −0.0006815 for the non-cancerous control sample (Figure 6A). The change was more extreme during the first hour of detection and then stabilized. During the first hour, the slope was positive for the metastatic sample, 0.0005643, and negative for non-metastatic and control samples, −0.002061 and −0.001428, respectively (Figure 6B).

### 3.5. Effect of pH on Detection

In addition, the pH of each original urine sample was measured to exclude the effect of pH on classification with sensors. The measured pH values varied from 5.2 to 7.2 (median of 5.9) for cancerous samples and from 5.7 to 6.7 (median of 6.2) for control samples. The median pH for the entire cohort was 6.07. There was no correlation between the pH of the samples and their clinical diagnoses. Statistically significant correlations were not found between the cancer and control samples (*p* = 0.0631), nor the metastatic cancer and non-metastatic samples (*p* = 0.8728) based on pH values. As a precaution, samples were diluted (1:50) for assays to minimize the pH variation between the samples and standardize the pH used in assays.

## 4. Discussion

The lack of accurate, non-invasive, and cost-effective methods to detect cancer, and especially its metastatic forms, early enough is a challenge for modern healthcare. New methods are urgently needed to detect, and furthermore, to identify new applicable biomarkers related to metastases at an early stage.

We chose one chemical sensor from our library based on previous knowledge of its capability to improve the detection of lethal prostate cancer [22]. Herein, it was chosen by a screen based on statistical differences between cancerous and control sample groups. We report, for the first time, this sensor’s capabilities to classify cancer in general using urine. Cancer was classified from control samples after 15 min of incubation by measuring the sensor with TRF. Detection was based on modulation in the chemical environment, which affects the capabilities of the label to emit TRF [24]. Additionally, we measured absorbance from the chemical sensor, with a more significant correlation to cancer already after 5 min of incubation. The factors interfering with TRF and absorbance measurements are likely partly the same. This chemical has been used for decades in the color-based “resazurin reduction test” for the detection of bacterial or yeast contamination in biological fluids and for sperm viability [25]. Likely, the color change happens when the oxidized, nonfluorescent form is reduced to the fluorescent form through cell activity and, most likely, metabolic oxygen consumption. However, it has been postulated to be reduced by mitochondrial enzymes [25], but it is not fully known whether the mechanistic pathways [26] are intracellular, on the membrane surface, or in the medium as a chemical reaction [25]. In turn, the protein concentration of the media has been noticed to have an effect on the change in form [27]. Later, this dye was applied to study the metabolic characteristics of cells [28] and tumor cells [29]. We applied a similar approach to a resazurin reduction test to cancer detection in urine. Our speculation is that the detection was based on the metabolism byproducts of cancerous cells or other molecules from a cancer-altered metabolism excreted to urine [4,5]. Therefore, we suggest that the detection is based on the interaction with these molecules, and the reaction takes place in the “medium”, i.e., in diluted urine.

Foremost, our main interest was to use M13 phages as a biorecognition element in the biosensor detecting metastatic cancer in urine. Various other practical applications in medicine, diagnostics, and engineering also have utilized the outstanding properties of phages [30,31,32,33]. In particular, their affinity-selection method can be utilized to discover new biomarkers for cancers in urine samples.

We deployed the advantageous properties of phages to our biosensor method in this study. We substantially refined our previously published biosensor development protocol [22] by preparing a large number of biosensor candidates targeting different urine sample pools, especially size-exclusion fractionated pools, instead of using a one pool to develop biosensors. We also used different absorbents, lignin and polystyrene, to bind the samples and increase the variety of different biomarkers to be introduced for phages in biopanning. The biosensor selection was carefully executed based on our extended understanding of the LC behavior of bifunctional phage biosensors [23]. The detection performance of biosensors was evaluated with absorbance measurements in various time points during a three-hour period to ensure that we found the best-performing candidate and measurement time point. These improvements in our system led to a phage biosensor that distinguished metastatic cancer from non-metastatic cancer and the control samples relatively well. Its calculated sensitivity was 70%, and its specificity was 79%. The accuracy remained nearly the same, even though the non-cancerous control samples were excluded from the analysis. Thus, the biosensor was reliably detecting the metastatic variants among non-metastatic cancerous samples and healthy control samples. The kinetic behavior of the biosensor with example samples demonstrated detection with the phage biosensor and gave one explanation for the result. In particular, within the first hour, the change in absorbance (slope) was in a different direction (slope was positive) with a metastatic sample than it was with non-metastatic ones (slopes were negative), and over measurement time, it was smaller than with others. This observation is most likely due to the LC behavior of the bifunctional phages. Non-metastatic samples without possible biomarkers can order themselves into the structurized phase with dye molecules, but with metastatic samples containing the biomarkers, the ordering is hindered.

We assume the detection was based on differences in urinary biomarkers between metastatic and non-metastatic samples. Either metastatic samples contained biomarkers related to the condition or lacked some biomarkers that were present in the non-metastatic samples. The rationale for using the NAP-10 column was that it allowed us to divide the samples into rough fractions according to their sizes. The column is typically used for DNA purification as it is excluded from the matrix and eluted first, and the small molecules were eluted last. We used the same column to search for the eluted fraction that provides the best phage binder classifying metastatic cancers. This fraction was located in the intermediate size of molecules that penetrated to the gel matrix. Furthermore, the simple method allowed for the removal of undesirable molecules that could disrupt the biopanning process. One example of biomarkers is glycosaminoglycans (GAGs), which are found in both plasma and urine. They have been used as potential biomarkers in MCEDs, with an AUC of approximately 0.80. MCED tests and their clinical applicability have recently gained attention [11]. With the reached sensitivities and specificities, our chemical sensor and biosensor could potentially be used as a complementary method with other screening platforms because the approach to finding potential biomarkers is quite different than that of other established methods. One clinically approved example combining multiple biomarkers is the 4Kscore test detecting high-grade prostate cancer from serum [34].

However, we do not yet specifically know the biomarkers interacting with our biosensor. This is, at the same time, both a disadvantage and an advantage. The method can be used to find completely new urinary biomarkers related to metastatic cancer or its development. Urine has gained attention lately as a new sample material for cancer screening [14]. Overall, we report here a completely new method to detect metastatic cancer using urine. The detection with a biosensor is based on the LC behavior of bifunctional phages [23]. We combine two attractive fields of biosensors, including phages [19,20] and LCs. In addition to phage benefits, LC sensors are also versatile and responsive to a wide range of stimuli. Both sensors are cost-effective and suitable for various applications [18].

Urinary pH varies between 4.5 and 8.0 because of abundant fluctuation in urine concentration caused by multiple factors, such as lifestyle and health conditions [35]. The lanthanide label used in the platform is sensitive to drastic acidic and alkalic conditions. Extreme pH causes fluorescence quenching and impedes detection with TRF. Even though samples were diluted (1:50) for assays to minimize pH variation between the samples and standardize the pH used in assays, the pH of each original urine sample was measured to exclude its effect on detection. There have been indications that acidic urinary pH can be associated with bladder [36] and prostate cancer [37]. However, our results did not have this indication in a cohort of mainly urological malignancies of the prostate and kidney. Moreover, our cohort included samples from two different sample cohorts. The samples were collected from two different hospitals in different regions of Finland, and they were sampled at different times. Older urine samples were collected in 2020 and stored at −80 °C after collection. The newer samples were collected during 2023. By combining these two sub-cohorts in this study, we obtained a strong indication that the sample storage in the freezer (years) or sampling in different locations did not affect the assay results. We checked these sample pH and storage effects on our system because the stability and environmental susceptibility are important factors to revise when developing new biosensors [18]. They must meet the requirements for clinical applications to have further clinical and commercial value.

Despite the strengths and promising indicative results in this study, it also has limitations that must be considered. Metastasizing causes systemic changes in the body because homeostasis is disturbed [38,39]. This disorder in homeostasis can lead to the uncontrolled leakage of simple ions and complex molecules from cells to extracellular space, eventually ending up in urine [40]. These metabolites can be single biomarkers or larger groups of molecules interfering with our detection result, or even causing a detection bias. Therefore, it is extremely important to identify the biomarkers and molecules interfering with our bifunctional phages in the biosensor. In a broader context, future research should focus on the spread of cancer in its early phases, before systemic changes occur in the human body. One research question would be to determine if we are measuring the overall weakening and disturbed homeostasis of the body or specific metabolic changes caused by metastatic cancer. Additionally, early detection is more appealing because early-stage cancers are generally more treatable. Further, the quality of the sample cohort may have affected the results because the number of control samples was smaller than the number of cancer samples. The control samples represented approximately 30% of the size of the whole cohort. Therefore, the control samples were not age- or biological sex-matched to the cancer samples. This led to uneven representation between different biological sexes and average age between the control group and cancerous groups. From the detection point of view, this study did not cover the classification of different cancers (PCa, KCa, others) included in the cohort. It would require developing biosensors targeting these specific cancers and their metastatic variants.

Hereby, our current research focuses on proving the indicative results reported in this paper. First, we are collecting a larger prospective case-control cohort with different metastatic cancers and corresponding control samples. The aim is to validate results with a larger cohort and to apply our platform to distinguish between different cancer types and the primary origins of metastases. Secondly, we will identify the detected metastasis-related biomarkers by sequencing the bifunctional phages and enriching biomarker–phage complexes and samples with mass-spectrophotometry. This could open many possibilities in urine biomarker research. Phage biosensors can detect known and yet unknown biomarkers from urine samples related to the disease itself, like metastatic cancer or its primary origin. Complementary biomarkers related to metastatic cancer could be found and have clinical potential. In particular, the early detection of metastatic cancers and follow-up treatment responses to support personalized treatment would be valuable tools for modern healthcare. Ultimately, the found biomarkers could be widely used in diagnostics, follow-up cancer treatment, and the development of new personalized treatments.

## 5. Conclusions

We report an improved biopanning protocol to develop bifunctional phage biosensors compared to our earlier work. Affinity selection toward size-exclusion fractionated urine yielded phage binders able to distinguish metastatic cancer from non-metastatic with a sensitivity of 70% and a specificity of 79%. Additionally, our chemical sensor interacting with cancer metabolism byproducts achieved a sensitivity of 71% and a specificity of 75% in general cancer detection using urine. These results underscore the potential of this approach in urinary biomarker research. Unknown cancer- and metastasis-related biomarkers may be found and enriched in urine with specifically selected phages. Currently, a study with a larger cohort of different urological cancers is underway. Moreover, we are expanding the study cohort prospectively to enhance our understanding of their clinical utility. The key points to study are how early the metastatic changes are visible in the patient’s urine as it is screened with our biosensors and what the limitations of the developed screening system are. Additionally, we plan to conduct in-depth investigations into the biomarkers associated with metastatic changes using mass spectrometry. Furthermore, we intend to sequence the bifunctional phages to gain deeper insights into their interactions with biomarkers. These efforts will contribute to a more comprehensive understanding of the biomarker landscape that is detected with our phage biosensors. Thereby, we can refine our biosensor technology for clinical needs.

## Figures and Tables

**Table 1:** Urine samples used in this study. Two cohorts, DEDUCER from HUS Helsinki University Hospital and BIOURICA from TYKS Turku University Hospital, were combined. Cancerous samples were collected from patients with metastatic and non-metastatic prostate, kidney, and other cancers (pancreas, bladder, penis, testis, retroperitoneal). Control samples were collected from healthy volunteers without previous cancer diagnoses.

Sample Type	Metastatic	Non-Metastatic	Total	Women	Men
Prostate cancer	12	17	29	0	29
Kidney cancer	11	4	15	2	13
Other cancers	5	4	9	1	8
Controls	n/a	n/a	21	2	19
Total	28	25	**74**	5 (7%)	69 (93%)

## Data Availability

The data presented in this study are available on request from the corresponding author (V.J.) The data are not publicly available due to commercial aims.
